# O Imenso Desafio de Buscar a Melhor Evidência

**DOI:** 10.36660/abc.20240106

**Published:** 2024-04-19

**Authors:** Luiz Maurino Abreu

**Affiliations:** 1 Hospital Federal dos Servidores do Estado Rio de Janeiro RJ Brasil Hospital Federal dos Servidores do Estado – Cardiologia, Rio de Janeiro, RJ – Brasil; 2 Estimulocor – Aval Clinica e Cardiológica Rio de Janeiro RJ Brasil Estimulocor – Aval Clinica e Cardiológica, Rio de Janeiro, RJ – Brasil

**Keywords:** Arritmias Cardíacas, Infecções por Coronavírus, Anticoagulantes, Fibrilação Atrial

Para gerar a melhor evidência científica, é crucial seguir métodos rigorosos de pesquisa, utilizar amostras representativas, aplicar análises estatísticas apropriadas e garantir a revisão por pares em publicações científicas respeitáveis. A transparência e replicabilidade também são fundamentais.^1^ Revisões sistemáticas e meta-análises têm como objetivo sumarizar um conjunto de evidências disponíveis para uma determinada questão e fornecer uma resposta de alta qualidade.^2^

Situações novas acrescem variáveis não analisadas anteriormente, e a pandemia de COVID-19 trouxe grandes questões. A urgência em encontrar tratamentos eficazes e desenvolver vacinas incentivou uma mobilização global da comunidade científica. Foram fatores que contribuíram: a emergência sanitária global, a mobilização internacional, fontes de financiamento prioritários, maior disponibilidade de redes de dados e alta tecnologia, que estimularam uma verdadeira corrida em busca de respostas. Essa convergência de fatores resultou em uma explosão de publicações, buscando melhor entender a fisiopatologia, opções de tratamento, medidas de prevenção e impactos socioeconômicos. Como seria de esperar, com a pressa, boa parte das publicações era de má qualidade, sendo que algumas geraram desinformação com graves consequências, pois muitas destas tiveram grande repercussão na mídia.^3^ No curso da pandemia inúmeras diretrizes foram geradas e alteradas.^4^ Houve discrepâncias notáveis entre as diferentes orientações em relação às recomendações sobre a gestão da COVID-19 no Brasil.^5^

O uso regular de anticoagulante oral (ACO) em pacientes com fibrilação atrial (FA), conforme seu perfil de risco, tem comprovado impacto na redução do acidente vascular cerebral tromboembólico e mortalidade, sendo indicação consagrada nas diretrizes com alto grau de evidência.^6^

O artigo de Landsteiner et al.,^7^ neste número dos ABC Cardiol, busca responder qual é a melhor conduta frente a um paciente portador de FA, em uso regular de ACO, e que é acometido de COVID-19. Os autores produzem uma cuidadosa meta-análise visando buscar a resposta quanto ao risco versus benefício do uso do ACO neste contexto clínico.

Metanálises de qualidade, em que se pode incluir estudos prospectivos randomizados, constituem o padrão ouro para a melhor evidência. No entanto, deve-se levar em conta os grandes desafios a serem superados para produzir uma meta-análise de qualidade com a seleção dos estudos a serem incluídos, identificar vieses potenciais, heterogeneidade, qualidade dos estudos, dados incompletos, viés de relato (publicações só com dados favoráveis), e a capacidade de generalização.^8^

Os autores^7^ fizeram busca sistemática no PubMed, Embase e Cochrane Library por estudos elegíveis desde o início da pandemia até dezembro de 2022, incluindo aqueles que compararam desfechos de COVID-19 em pacientes com e sem anticoagulação oral crônica prévia para FA. O trabalho de seleção, empregou protocolo rigoroso buscando publicações com maior qualidade e menor chance de vieses, fundamental tratando-se de estudos observacionais. A descrição da "Análise Estatística" presente no artigo,^7^ dá a noção de quão complexo é o processo, com inúmeras ferramentas sendo utilizadas para buscar reduzir as potenciais limitações e assim, a melhor evidência. Inicialmente foram selecionados 596 estudos, com 493 excluídos, 26 revisados e, só considerados 10 para análise. Apesar do robusto número de casos incluídos, a heterogeneidade sempre foi elemento a considerar.

Para avaliar o risco de vieses no material selecionado, os autores usaram a ferramenta ROBINS-I ( *Risk of Bias in Non-randomized Studies of Interventions* ). Suas limitações incluem dependência da qualidade dos relatos, desafios na categorização de viés e a necessidade de julgamento subjetivo por parte dos avaliadores. Além disso, a ROBINS-I pode não abordar completamente todas as fontes de viés potencial em certos contextos. Esse é um dos aspectos ressaltado no próprio artigo nas limitações do estudo.^9 , 10^

Com todo isso, os autores na discussão, reconhecem não haver total segurança dos resultados, principalmente pelo desconhecimento das condições e quais foram os impedimentos para tratamento hospitalar, o que só valoriza o trabalho agora publicado.

A conclusão está de acordo com a plausibilidade científica mais forte: a anticoagulação reduz o risco nos pacientes com FA.

Como sempre, a melhor decisão será aquela tomada caso a caso, onde riscos versus benefícios serão avaliados e compartilhados com o paciente, de acordo com as evidências disponíveis.
