# Supplementary material for: O Imenso Desafio de Buscar a Melhor Evidência
Source: Arq Bras Cardiol. 2024 Apr 19;121(3):e20240106. [Article in Portuguese] doi: 10.36660/abc.20240106 (PMC11081097; doi:10.36660/abc.20240106)
Supplement: Translation

# Translation: The Immense Challenge of Searching for the Best Evidence

**DOI:** 10.36660/abc.20240106i

**Authors:** Luiz Maurino Abreu

**Affiliations:** 1 Estimulocor – Aval Clinica e Cardiológica Rio de Janeiro RJ Brazil Hospital Federal dos Servidores do Estado – Cardiologia, Rio de Janeiro, RJ – Brazil; 2 Estimulocor – Aval Clinica e Cardiológica Rio de Janeiro RJ Brazil Estimulocor – Aval Clinica e Cardiológica, Rio de Janeiro, RJ – Brazil

**Keywords:** Arrhytmias, Cardiac, Coronavirus, Infections, COVID-19, Anticoagulants, Atrial Fibrillation

To generate the best scientific evidence, it is crucial to follow rigorous research methods, use representative samples, apply appropriate statistical analyses, and ensure peer review in reputable scientific publications. Transparency and replicability are also key.^1^Systematic reviews and meta-analyses aim to summarize a body of available evidence for a given question and provide a high-quality answer.^2^

New situations add variables not previously analyzed, and the COVID-19 pandemic has raised big questions. The urgency to find effective treatments and develop vaccines has encouraged a global mobilization of the scientific community. Contributing factors were: the global health emergency, international mobilization, priority funding sources, greater availability of data networks, and high technology, which stimulated a real race in search of answers. This convergence of factors resulted in an explosion of publications, seeking to better understand the pathophysiology, treatment options, prevention measures, and socioeconomic impacts. As would be expected, in the rush, most of the publications were of poor quality, some of which generated misinformation with serious consequences, as many of these had great repercussions in the media.^3^During the course of the pandemic, numerous guidelines were generated and changed.^4^There were notable discrepancies between different guidelines regarding recommendations on the management of COVID-19 in Brazil.^5^

The regular use of oral anticoagulants (OAC) in patients with atrial fibrillation (AF), depending on their risk profile, has proven to have an impact on reducing thromboembolic stroke and mortality, being an indication enshrined in guidelines with a high degree of evidence.^6^

The article by Landsteiner et al.,^7^in this issue of the ABC Cardiol, seeks to answer what is the best approach for a patient with AF, who regularly uses OCP, and who is affected by COVID-19. The authors produce a careful meta-analysis aiming to find the answer regarding the risk versus benefit of using OAC in this clinical context.

Quality meta-analyses, which can include prospective randomized studies, constitute the gold standard for the best evidence. However, one must take into account the major challenges to be overcome to produce a quality meta-analysis with the selection of studies to be included, identify potential biases, heterogeneity, quality of studies, incomplete data, reporting bias (publications only with favorable data), and the ability to generalize.^8^

The authors^7^systematically searched PubMed, Embase, and Cochrane Library for eligible studies from the start of the pandemic until December 2022, including those that compared COVID-19 outcomes in patients with and without prior chronic oral anticoagulation for AF. The selection work used a rigorous protocol seeking publications with higher quality and less chance of bias, which is essential in the case of observational studies. The description of the "Statistical Analysis" present in the article^7^gives the notion of how complex the process is, with numerous tools being used to seek to reduce potential limitations and thus, the best evidence. Initially, 596 studies were selected, with 493 excluded, 26 reviewed, and only 10 considered for analysis. Despite the robust number of cases included, heterogeneity was always an element to consider.

To assess the risk of bias in the selected material, the authors used the ROBINS-I (Risk of Bias in Non-randomized Studies of Interventions) tool. Its limitations include dependence on the quality of reports, challenges in categorizing bias, and the need for subjective judgment on the part of evaluators. Additionally, ROBINS-I may not fully address all sources of potential bias in certain contexts. This is one of the aspects highlighted in the article itself in the limitations of the study.^9 , 10^

With all this, the authors in the discussion recognize that there is no complete certainty of the results, mainly due to the lack of knowledge of the conditions and what were the impediments to hospital treatment, which only adds value to the work now published.

The conclusion is in line with the strongest scientific plausibility: anticoagulation reduces the risk in patients with atrial fibrillation.

As always, the best decision will be made on a case-by-case basis, where risks versus benefits will be assessed and shared with the patient, according to the available evidence.
